# Managing low–acuity patients in an Emergency Department through simulation–based multiobjective optimization using a neural network metamodel

**DOI:** 10.1007/s10729-024-09678-3

**Published:** 2024-06-10

**Authors:** Marco Boresta, Tommaso Giovannelli, Massimo Roma

**Affiliations:** 1 Institute for System Analysis and Computer Science “A. Ruberti”, National Research Council of Italy, via dei Taurini, 19, Rome, 00185 Italy; 2 Department of Industrial and Systems Engineering, Lehigh University, 200 W Packer Ave, Bethlehem, PA 18015 USA; 3 Department of Computer, Control and Management Engineering “A. Ruberti”, SAPIENZA – University of Rome, via Ariosto 25, Rome, 00185 Italy

**Keywords:** Emergency department fast-track, Discrete event simulation, Simulation-based optimization, Metamodel, Neural network, Multiobjective optimization, Machine learning

## Abstract

This paper deals with Emergency Department (ED) fast-tracks for low-acuity patients, a strategy often adopted to reduce ED overcrowding. We focus on optimizing resource allocation in minor injuries units, which are the ED units that can treat low-acuity patients, with the aim of minimizing patient waiting times and ED operating costs. We formulate this problem as a general multiobjective simulation-based optimization problem where some of the objectives are expensive black-box functions that can only be evaluated through a time-consuming simulation. To efficiently solve this problem, we propose a metamodeling approach that uses an artificial neural network to replace a black-box objective function with a suitable model. This approach allows us to obtain a set of Pareto optimal points for the multiobjective problem we consider, from which decision-makers can select the most appropriate solutions for different situations. We present the results of computational experiments conducted on a real case study involving the ED of a large hospital in Italy. The results show the reliability and effectiveness of our proposed approach, compared to the standard approach based on derivative-free optimization.

## Highlights

- Our study focuses on fast-tracks for low-acuity patients, commonly used to reduce ED overcrowding.
- We provide a mathematical formulation of the problem with the aim of minimizing patient waiting times and ED operating costs.
- The approach we propose provides ED managers with a decision support system for determining the best strategy to adopt.
- This system enables ED managers to define the optimal resource allocation of minor injuries ED units, where low-acuity patients can be diverted via a fast-track.

## Introduction

Emergency Medical Services play a central role among healthcare services as they are devoted to providing timely medical treatments to people in need of urgent care [6]. The Emergency Department (ED) is usually considered one of the most important emergency services, as hospital EDs typically provide the first care to urgent patients transported by ambulance or arriving autonomously. Unfortunately, the worldwide phenomenon of overcrowding may significantly affect the quality and promptness of the care delivered. This phenomenon often results in long waiting times that may endanger the lives of critical patients by increasing the risk of deteriorating health conditions. Besides visit and treatment delays (especially for low-acuity patients), overcrowding generates unpleasant phenomena, such as an excessive number of patients in the ED, patients treated in the hallways, an increasing number of patients who leave without being seen (LWBS), ambulance diversion, reduced patient satisfaction, and overloaded ED staff. The increase in patient mortality can also be directly attributed to the ED overcrowding problem [25, 68]. The causes of this issue can be traced back to both exogenous factors, such as flu season and requests for non-urgent visits, and endogenous factors, such as shortages in ED internal resources (observation units, beds) and understaffing.

Several Key Performance Indicators (KPIs) can be adopted to assess ED performance (see the recent paper [77]) and, in particular, to estimate the overcrowding level. These include commonly used metrics such as the Length Of Stay (LOS) in the ED, the waiting time before the first medical visit, also known as Door-To-Doctor Time (DTDT), and the percentage of LWBS patients. Moreover, some more sophisticated measures have been proposed to quantify ED crowding and staff workload, aiming to prevent critical conditions by implementing proactive solutions. Examples of such measures include NEDOCS, READ, EDWIN, and the Work Score [1, 10, 79–81]. However, studies have shown that none of these methods are consistently effective in providing timely warnings while maintaining low rates of false alarms [41].

Efficient management of both physical and human re-sources in an ED, along with the adoption of strategies to improve patient flow, are essential components of any effort to address the overcrowding problem [3]. In particular, specific Fast-Tracks (FTs) are often implemented to alleviate this phenomenon. FT systems were introduced in the late 1980s in North American hospitals and later in the United Kingdom and Australia. Today, they are adopted in hospitals around the world, and the advantages of using FTs have been highlighted in many case studies (see, e.g., the review article [84]). A significant reduction in the ED LOS is usually observed, along with a decrease in the percentage of LWBS patients and the average number of patients in the ED. As a direct consequence, providing more timely and higher-quality care leads to improved patient satisfaction and safety. This motivates the rapid adoption of FT systems, which are now prevalent in most EDs.

In this paper, we focus on *FTs for low-acuity patients*, which constitute a dedicated pathway for individuals with non-urgent complaints and uncomplicated diseases. These patients are identified by the triage nurse and directed via the corresponding FT to a specific unit or area known as the *Minor Injuries Unit* (MIU). In the MIU, patients receive treatment and can often be discharged relatively quickly. This unit typically requires specific resources not shared with other ED units and is staffed with dedicated senior medical and nursing personnel who can make swift discharge decisions. Operating hours for the MIU are usually determined by ED managers and are restricted to specific daytime hours, rather than offering 24-hour service. The allocation of resources in the MIU is closely related to the percentage of patients directed to it via the corresponding FT by the triage nurse. The operating costs of the MIU depend on the allocated resources and its operating hours.

Given the substantial variability in operations, the de-cision-making process for designing the FT system and the MIU is highly complex. Thus, providing ED managers with a Decision Support System (DSS) could prove highly beneficial. Such a system would optimize KPIs by facilitating strategic patient diversion via the FT system and ensuring efficient resource allocation for the MIU. As far as we are aware, the adoption of such a DSS system is rare in practice, and managers typically rely on their own experience to make empirical decisions regarding the allocation of resources in the MIU and its operating hours. For instance, they might choose to align the MIU’s opening hours with the highest overall daily influx of ED patients and they might consider closing the MIU on Sundays due to lower patient volumes or staffing constraints on weekends. However, the effectiveness of the MIU is closely related to the percentages of patients diverted to it, making these percentages fundamental control variables in the problem. Consequently, the role of the triage nurse is crucial from a practical standpoint. A conservative approach by the triage nurse, aimed at retaining most patients within standard ED pathways, would undermine the goal of an FT system. Therefore, when implementing an FT for low-acuity patients, an enhanced triage process becomes desirable. It is important to note that the same reasoning extends to specialist clinical pathways, such as those for ophthalmologists, orthopedists, and various other specialties. These pathways are commonly utilized in EDs to directly guide patients with specific pathologies, identified during triage, to specialized units.

In this paper, we address the optimal resource allocation problem in the MIU as part of a strategy to manage ED low-acuity patients within an FT system. We formulate this problem using a multiobjective optimization formulation to minimize potentially conflicting goals, such as the expected value of the overall patient waiting time (specifically, the DTDT) and MIU operating costs (measured in terms of unit working hours and the number of rooms used in the MIU). These goals can be conflicting because reducing patient waiting time may require increasing the MIU working hours and the number of rooms, thus leading to higher operating costs. Note that our approach is not restricted to these objectives and allows for the consideration of any other KPIs, such as LOS and the percentage of LWBS patients.

Addressing the multiobjective optimization problem we aim to solve poses a significant challenge, as it cannot be formulated analytically due to the unavailability of the KPIs of interest in closed form. Indeed, due to the intricate nature of the processes within an ED, there are typically no tractable analytical models available to compute the KPIs as outputs of functions that take input variables representing ED settings. Therefore, it is necessary to resort to a simulation model that simulates the processes within an ED, obtaining the values of the KPIs as outputs of simulation runs. As a result, the problem falls into the domain of Simulation-Based Optimization (SBO), a well-established field that combines Optimization and Simulation techniques. In SBO problems, which are widely recognized as challenging (see, e.g., [4, 30, 31, 34]), function evaluations are associated with “measurements” from experimental simulations, and the functions can be very expensive to evaluate due to the complexity of the simulation. Moreover, since simulation responses (outputs) exhibit noise, the adopted optimization techniques need to be robust enough to converge despite this noise in function evaluations. Since function derivative information is unavailable, derivative-free optimization (DFO) or black-box optimization methods must be used [7, 16, 19, 26, 33, 54, 57, 58]

To address the significant challenges posed by SBO, we propose using a metamodeling approach that uses an Artificial Neural Network (ANN) to compute the KPIs of interest. In particular, the key idea is to construct a machine learning model that “mimics” the simulation model representing patient flow through the ED. This enables the inexpensive evaluation of the expected value of the overall patient DTDT. An accurate machine learning model that captures the relationships between the input and the output variables can be efficiently used as an alternative to the simulation model. In particular, we adopt a Multi-Layer Perceptron architecture for its well known universal approximation properties [20, 55].

The approach we propose has the following advantages:

- the only time-consuming phase concerns the generation of the dataset used to train the neural network;
- it determines optimal solutions of an SBO problem through powerful gradient-based methods;
- it generates Pareto optimal solutions of the multiobjective optimization problem with a better coverage of the Pareto front;
- parallel computing techniques can be used to further improve the overall efficiency.

We performed an extensive experimentation of our approach on a real case study, namely, the ED of a large hospital in Rome (Italy), where an FT is currently used to possibly divert low-acuity patients to the MIU. We formulate the optimal resource allocation of this MIU as a bi-objective mixed integer SBO problem and we determine Pareto optimal points by using the weighting method. We emphasize the importance of providing ED managers with a set of optimal solutions rather than a single one. This approach allows them to select the most suitable solution based on their specific needs. The solution points obtained through our experimentation clearly indicate that, considering the criteria we adopted, the current ED setting is far from optimal. Improvements can be made both in terms of the overall patient DTDT and the MIU operating costs.

To assess the reliability and efficiency of our proposed ANN-based metamodeling approach, we conducted a comparison with the results obtained using the standard procedure in SBO, which involves the use of a derivative-free optimization method directly combined with a simulation model. Despite adopting a highly efficient derivative-free optimization algorithm recently proposed in the literature, our comparison clearly demonstrates the superiority of the ANN-based metamodeling approach, particularly in terms of the Pareto front coverage. Specifically, our approach demonstrates a tendency to cover a wider range of trade-off solutions, which is a crucial advantage in our context. This superiority can be attributed to the significant computational burden required to directly evaluate a black-box objective function through simulation model runs, which exhausts the computational budget before obtaining a sufficient number of Pareto optimal solutions.

The organization of the paper is as follows: Section 2 provides a literature review on the use of FT systems and the simulation-based metamodeling approach in ED resource planning. Section 3 presents the formal statement of the MIU optimal resource allocation problem, while Section 4 describes the methodologies considered for addressing it. Our proposed ANN-based metamodeling approach is detailed in Section 5. Section 6 presents a real case study, and the associated MIU management problem is outlined in Section 7. Experimental results on the case study are provided in Section 8, along with some findings from sensitivity analysis experiments. Finally, concluding remarks are presented in Section 9.

## Literature review

The literature on ED overcrowding is extensive. Readers are directed to the review paper [40] (and the references therein), which highlights the main causes and effects of overcrowding, along with potential solutions. Other papers addressing the overcrowding phenomenon include [21, 44, 63–65, 82]. FT systems and enhanced triage models are commonly employed to address overcrowding, as noted in [3]. The review article [84] discusses strategies such as team triage, streaming, and fast-tracking, which have been proven to reduce ED overcrowding. Additionally, the article explores some ideal models for the patient journey within an ED.

Using an FT for patients with low-acuity illnesses and injuries is a strategy analyzed in many studies aiming at assessing its impact on reducing patient waiting times and, accordingly, the ED overcrowding [18, 45, 46, 62, 64, 69]. In particular, in [64], the authors report that the expected benefits of using FT systems are observed in every ED, regardless of the specific case studies considered. In [69], the authors show that reducing the number of low-acuity patients does not significantly affect the waiting times of the high-acuity patients when the resources in charge of the visit and treatment of low-acuity patients are dedicated. Contrarily, when such resources are shared with critical patients, a worsening in the total average waiting times of the most urgent patients may be experienced [51]. The authors in [51] show that most benefits of the FT system are observed in EDs with a considerable number of urgent patients, as the improvement in terms of waiting time is higher when the low-acuity patients can bypass a larger number of patients. In the case study discussed in [78], the effects of FT systems on the ED performance of Australian hospital EDs are thoroughly analyzed. The study concludes that FTs enable effective care for patients with minor illnesses without negatively affecting treatments for other non-FT patients. Additionally, [17] reports the results of another case-controlled study, clearly highlighting several benefits of adopting an FT.

Among the methodologies adopted, Discrete Event Simulation (DES) models (see, e.g., [9, 27, 35, 45, 50, 52, 83, 89]) and Agent-Based Simulation (ABS) (see, e.g., [5, 46, 56]) are frequently used to study patient flow through an ED. For a comprehensive review of simulation modeling applied to EDs, we refer the reader to the recent paper [67] and the references therein. Moreover, some papers dealing with ED management have proposed the use of SBO approaches, combining a simulation model with an optimization algorithm [2, 13, 23, 24, 28, 36, 37, 53, 75, 76, 88, 90]. However, most of the published papers on FTs only use simulation modeling to assess their effectiveness. Sometimes, simulation is combined with other techniques (see, e.g., [39], where simulation is combined with the system dynamics approach), but to the best of our knowledge, papers using optimization are still very few. Indeed, many papers examine the impact of the adoption of FT strategies through scenario-based analyses, and only a few focus on identifying the settings that provide effective diversion policies by optimizing KPIs. A recent paper adopting the latter approach is [71], where a multi-criteria method is proposed to determine the best configuration for an FT system in terms of five performance indicators.

The use of metamodel-based SBO is very limited in healthcare. A recent systematic literature review [72] reports only six papers in the healthcare field, with four of them (i.e., [15, 86, 87, 89]) applying a metamodel-based SBO approach to ED resource planning, albeit none focusing on FTs. In particular, [89] proposes the combined use of SBO with a metamodel to determine optimal ED resource allocation, considering both budget and capacity constraints, with the goal of minimizing the total average patient waiting time. The computationally expensive simulation model is replaced by a suitable metamodel, chosen among several alternatives. The authors showed that for their case study, an ANN-based metamodel exhibited superior performance compared to two other metamodels considered. In the ED management context, another paper replacing a DES model with an ANN-based metamodel is [85], where a resource allocation problem is considered by analyzing the impact of human errors caused by the increase in ED workload.

## Statement of the MIU optimal resource allocation problem

In order to define the problem we are addressing, let us first briefly review some structural and operational elements that characterize an ED. The *triage process* is the initial activity a patient undergoes upon arriving at the ED. During this phase, a triage nurse conducts an initial assessment and assigns a *severity tag* to each patient. Such assignment corresponds to prioritizing patients based on their acuity level. Various scales can be adopted; for instance, the five-level Emergency Severity Index (ESI) commonly used in the USA stratifies patients into five groups ranging from 1 (most urgent) to 5 (least urgent) [32]. For simplicity, tags can often be associated with colors, such as red, yellow, green, and white (listed in order of decreasing severity), which are used in four-level scales. National Health Systems guidelines usually provide a threshold value that should not be exceeded by the average patient waiting time (i.e., DTDT) and stay time (i.e., LOS), for each triage tag. We denote the set of severity tags by $\mathcal{T}$.

From a structural point of view, an ED typically consists of several *units* (e.g., surgical unit, resuscitation area, etc), which are ED areas or rooms to which patients are directed post-triage based on their pathology. We will indicate the set of ED units by *U*. More precisely, for each severity tag $t\in \mathcal{T}$, we denote by *U*(*t*) the set of ED units where a patient tagged with severity tag *t* can be directed. ED units are usually equipped differently, and patients can only be assigned to one of them based on their triage tag.

In our paper, we focus on FT systems within EDs that offer prompt and effective care to low-acuity patients. Using an FT involves diverting such patients towards a dedicated ED unit (i.e., the MIU) to expedite their visit, treatment, and discharge, thus preventing excessive crowding in the standard clinical pathways of the ED. This approach is expected to result in an overall decrease in the number of patients in the ED, DTDT, and LOS. Two key issues underlie the success of such an FT strategy:

- suitable allocation of physical and human resources for the MIU, along with a proper operating schedule;
- enhanced triage, facilitating the prompt identification of patients with low-acuity illnesses and injuries for direct transfer to the MIU via the FT.

The first issue concerns the allocation of resources within the MIU. Typically, the MIU comprises a set of rooms equipped with armchairs, stretchers, and beds, where dedicated personnel (physicians and nurses) attend to and treat patients. Additionally, since the MIU usually operates during the day, its opening and closing times must be predetermined for each day within a specified time period (typically on a weekly basis). The second issue pertains to the proportion of patients directed to the FT by the triage nurse. While the nurse’s decision is primarily based on the clinical assessment of patients, an overly cautious approach by the triage nurse may result in a low percentage of patients being diverted to the MIU, rendering the FT system ineffective.

Now, we can formally introduce the problem. Let $D = \{1, \ldots , n\}$ represent the set of days within the chosen time period (for example, $D = \{1, \ldots , 7\}$ if a weekly schedule is adopted). The MIU optimal resource allocation problem consists of deciding the number of rooms to be open and their operating hours for each day within the period *D*. It is important to note that, due to fluctuations in patient flow, the required number of MIU rooms to be open, as well as the opening and closing times, may vary from day to day.

Optimizing the opening and closing times of the MIU arises from the need to efficiently allocate limited resources while balancing resource costs and patient waiting times. The goal is to reduce patient waiting times while minimizing total personnel costs at the MIU. Therefore, in the following problem formulation, we consider the MIU opening and closing times as decision variables. Note that this choice is reasonable only if the MIU personnel can flexibly adjust their shifts to accommodate the chosen opening and closing times. In particular, in the case study discussed in Section 6, the decision to optimize the MIU opening and closing times stemmed directly from a request by the ED management.

### The decision variables

To formulate the problem, we introduce the following decision variables:

- let $x_d \in \mathbb {Z}^+$ be the *opening time* of the MIU on day $d \in D$;
- let $y_d \in \mathbb {Z}^+$ be the *closing time* of the MIU on day $d \in D$;
- let $r_d \in \mathbb {Z}^+$ be the *number of rooms* used in the MIU on day $d\in D$;
- let $z \in \mathbb {R}$ be the *percentage of patients* assigned to the MIU during its working hours.

Therefore, we have three *n*-dimensional integer valued vector variables, namely $\textbf{x}=(x_1, \ldots , x_n)^\top$, $\textbf{y}=(y_1, \ldots , y_n)^\top$, and $\textbf{r}=(r_1, \ldots , r_n)^\top$, and the percentage real value *z*. Hence, $(\textbf{x}, \textbf{y}, \textbf{r}, z)\in \mathbb {Z}^n \times \mathbb {Z}^n \times \mathbb {Z}^n \times \mathbb {R}$ represents the MIU setting for the time period *D*. Note that variables $x_d$ and $y_d$ are assumed to be integer since opening and closing times usually refer to exact hours (e.g., 8.00 a.m. – 4.00 p.m.).

The rationale behind the choice of considering the percentage of patients that are assigned to the MIU as a decision variable is as follows: although the triage nurse makes decisions based on the seriousness of the patient’s health, including this percentage as a decision variable aims to demonstrate the potentially significant improvement that would be obtained if the number of patients assigned to the MIU were increased. The hope is that showing the potential benefits obtained from experimental results can encourage the nurse to send a larger number of patients to the MIU, thereby reducing the workload of the other ED units. However, it is important to note that some scenarios may be idealistic, as the nurse’s choice is strictly related to the severity of the patient’s condition.

Note that a working day can be divided into several time slots, and different percentages of patients diverted to the MIU could be chosen for each time slot. This simply requires introducing variables (such as $z_i$) for each time slot.

### The objective functions

We can now define the objective functions of the problem under consideration. First, we introduce the concept of sample response functions, which represent the relationships between inputs and outputs of a simulation model. Such functions are used to evaluate the KPIs of interest that characterize the objective functions.

Given a time period *D*, for each tag $t\in \mathcal{T}$ and unit $u\in U(t)$, we denote a *sample response function* by $F^{t u}(\textbf{x}, \textbf{y}, \textbf{r}, z; \xi (\omega ))$, where $(\textbf{x}, \textbf{y}, \textbf{r}, z)$ represents the MIU setting and $\xi (\omega )$ is a random vector defined on a probability space representing the randomness. Specifically, random realizations $\xi _i$ of $\xi (\omega )$ correspond to different patient flows through the ED. When a simulation model is used to represent ED patient flow, a single simulation run evaluates $F^{t u}(\textbf{x}, \textbf{y}, \textbf{r}, z; \xi _i)$ for a random realization $\xi _i$. In this framework, sample response functions are commonly adopted to compute waiting times or stay times in the ED, such as the DTDT or LOS, or to compute certain counters, such as the number of patients in the ED or the number of patients waiting in a queue. Typically, objective functions are defined by the expected value of a sample response function, i.e., $\mathbb {E}\left[ F^{t u}(\textbf{x},\textbf{y},\textbf{r},z;\xi (\omega ))\right]$, where $\mathbb {E}[\cdot ]$ denotes the expected value taken with respect to the probability distribution of $\xi (\omega )$. Note that a sample response function is not available in closed form because the analytical relationship between inputs and outputs of the simulation model is unknown. As a result, the corresponding objective functions do not have an explicit form either.

We can now define the objective functions of the MIU optimal resource allocation problem. The first objective function computes the expected value of the overall patient waiting time over the time period *D*, namely,

1 $$\begin{aligned} \begin{array}{l} f_1(\textbf{x},\textbf{y},\textbf{r},z) = \\ \displaystyle \sum _{t \in \mathcal{T}}\alpha _t\sum _{u \in U(t)}\beta _u \,\, \mathbb {E}\left[ DTDT^{tu}(\textbf{x},\textbf{y},\textbf{r},z;\xi (\omega )) \right] , \end{array} \end{aligned}$$

where $\alpha _t>0$ and $\beta _u>0$ are scalars, and $DTDT^{tu}(\textbf{x},\textbf{y},\textbf{r},$ $z;\xi (\omega ))$ is the sample response function representing the DTDT of a *t*-tagged patient assigned to the ED unit *u*, given the MIU setting during the period *D* as $(\textbf{x},\textbf{y},\textbf{r},z)$. The second objective function counts the MIU working hours over the time period *D*, namely,

2 $$\begin{aligned} f_2(\textbf{x},\textbf{y}) = \sum _{d\in D} \gamma _d (y_d-x_d), \end{aligned}$$

where $\gamma _d>0$ are scalars. Finally, the third objective function calculates the number of MIU rooms open daily over the time period *D*, namely,

3 $$\begin{aligned} f_3(\textbf{r}) = \sum _{d\in D} \delta _d r_d, \end{aligned}$$

where $\delta _d>0$ are scalars. The scalars in Eqs. 1, 2, and 3 are introduced to accommodate potential variations in weighting among the individual terms of the objective functions.

### The constraints

Simple box constraints must be imposed on the variables, as follows

4 $$\begin{aligned} \begin{array}{c} l_{x_d} \le x_d \le u_{x_d} \\ l_{y_d} \le y_d \le u_{y_d} \\ l_{r_d} \le r_d \le u_{r_d} \\ l_z \ \, \le \; z \, \le u_z, \end{array} \end{aligned}$$

for all $$d \in D$$, where $$l_{x_d}$$, $$l_{y_d}$$, $$l_{r_d}$$ and $$u_{x_d}$$, $$u_{y_d}$$, $$ u_{r_d}$$ are non-negative integer prefixed lower and upper bounds, respectively, and $$l_z$$, $$u_z$$ are real values in [0, 100]. The practical selection of the upper bound $$u_z$$ is a critical choice. Accurate tuning of this value is necessary to prevent unrealistic scenarios. Note that the value of $$u_z$$ depends on the conservative approach of the triage nurse in directing patients to the MIU via the FT and also on the patient mix in the ED (available from historical data), since not all patients are eligible for transfer to the MIU. Additionally, if multiple variables $$z_i$$ are introduced to take into account different time slots, it may be appropriate to define a different bound for each of them.

Moreover, we must consider the constraints

5 $$\begin{aligned} y_d-x_d \ge h_d, \end{aligned}$$

for all $d \in D$, where $h_d \ge 0$ is an integer value that serves as the lower bound for the daily minimum number of hours that the MIU must be open. It is necessary to impose such a requirement to ensure that the MIU operates for a sufficient duration each day, particularly if a weekly threshold for the total number of operating hours is desired.

An additional constraint imposes a lower bound on the overall number of MIU working hours in the time period *D*, namely,

6 $$\begin{aligned} \sum _{d\in D} \left( y_d-x_d \right) \ge g, \end{aligned}$$

where *g* is a prefixed non-negative integer. This constraint ensures that the MIU remains open for an adequate duration throughout the entire period *D*, providing consistent service.

### The multiobjective simulation-based optimization problem

The MIU optimal resource allocation problem can be formally stated as

7 $$\begin{aligned} \begin{array}{rl} \text {min} &{} \left( f_1(\textbf{x},\textbf{y},\textbf{r},z) , f_2(\textbf{x},\textbf{y}) , f_3(\textbf{r}) \right) ^\top \\ \text {s.t.} &{} {(\textbf{x}, \textbf{y}, \textbf{r}, z)\in \mathcal{F}}, \end{array} \end{aligned}$$

where $\mathcal{F}$ represents the feasible set, i.e., the set of points satisfying all the constraints defined in Section 3.3. Problem Eq. 7 constitutes a multiobjective mixed integer constrained optimization problem.

It is important to note that the number of MIU working hours directly influences the MIU operating costs: the longer the MIU rooms remain open, the higher the operating expenses. Therefore, since our aim is to minimize both the expected value of the overall patient waiting time and the MIU operating costs, we formulate the optimization problem as a simultaneous minimization of the functions $f_1$, $f_2$, and $f_3$. This results in a problem with conflicting objectives, where the goal is to find a trade-off between reducing operating expenses and ensuring timely treatments for patients based on their urgency level.

A significant challenge arises when addressing problem Eq. 7. As previously noted, the intricate and stochastic nature of processes within the ED precludes the use of analytical models, necessitating the adoption of simulation models. Among these, DES models stand out for their flexibility and robustness in modeling patient flow through an ED. By leveraging this approach, we can construct and validate an ED DES model that allows us to evaluate the sample response function associated with the overall patient waiting time through simulation runs. Therefore, the problem we are considering is a *Mutiobjective (mixed integer) Simulation-Based Optimization (MOSBO)* problem.

## Methodology

In this section, we describe the methodologies we use to solve the MOSBO problem stated in Eq. 7.

### Sample average approximation

The most commonly used technique for addressing SBO problems is the *sample average approximation*, also called *sample path method* (see, e.g., [47, 49]). This approach involves approximating the expected values of a sample response function with deterministic sample averages. Specifically, the expected value of the sample response function $F^{tu}(\cdot )$ is approximated by a deterministic realization of the underlying stochastic function:

8 $$\begin{aligned} \begin{array}{l} \mathbb {E}\left[ F^{tu}(\textbf{x},\textbf{y},\textbf{r},z;\xi (\omega ))\right] \approx \\ \displaystyle \frac{1}{N} \sum _{i=1}^N F^{tu}(\textbf{x},\textbf{y},\textbf{r},z;\xi _i)= \widehat{F}_N^{tu}(\textbf{x},\textbf{y},\textbf{r},z), \end{array} \end{aligned}$$

where $\{\xi _1, \ldots \xi _N\}$ is an *independent and identically distributed sample*.

Given a sample $\{\xi _1, \ldots \xi _N\}$, the sample average response function $\widehat{F}_N^{tu}( \cdot )$ is deterministic, allowing for the application of deterministic (Derivative-Free/Black-Box) optimization algorithms for its minimization. In the DES modeling framework, each $\xi _i$ corresponds to the output of a single replication in a simulation run, and the average in Eq. 8 is computed over *N* independent replications. Therefore, each evaluation of the function $\widehat{F}_N^{tu}( \cdot )$ may require a significant amount of computational time. Hence, optimization methods that require a very large number of function evaluations to generate a good solution (such as standard heuristics and/or Derivative-Free methods) may not be practical in this context.

### Response surface methodology

The necessity of running a DES model to evaluate objective functions implies that the direct application of an optimization algorithm typically results in a time-consuming procedure. The required computational effort depends on the complexity of the simulation model and the length and number of replications for each run. Therefore, the standard procedure of solving SBO problems by directly combining a simulation model with an optimization algorithm may demand extended CPU times.

To overcome this drawback, *Response Surface Methodology (RSM)* is often used (see, e.g., [34, Chapter 4] and [4, 30]). RSM constructs an approximate functional relationship between the input variables and the output objective function. In particular, RSM involves building a *metamodel* (also known as a *surrogate model*) that represents an approximation of the objective function obtained by simulating the system at a finite number of sampled points across the entire parameter space. The hope is that the metamodel will provide a good approximation of the function, which is not available in closed form, once the metamodel parameters are accurately estimated. Then, the approximate underlying objective function can be evaluated easily and inexpensively, enabling the adoption of powerful derivative-based optimization procedures. For further information on the use of metamodels in SBO, we refer the reader to [8].

Different RSM approaches vary based on the nature of the functional form used for approximation. The most commonly used functional forms are based on polynomial response surface approximations. Other metamodeling approaches use splines, radial basis functions, Kriging models, or neural networks. In our paper, we adopt a neural network-based metamodeling approach for solving the MOSBO problem stated in Eq. 7, as detailed in the next sections. This approach is also referred to as the *neuro-response surface method* (see [34, Chapter 4]).

## The proposed Artificial Neural Network-based metamodeling approach

We introduce the use of an Artificial Neural Network (ANN) to efficiently solve the MOSBO problem stated in Eq. 7, specifically, to determine its Pareto optimal points. The key idea is to construct a machine learning model that “mimics” the DES model, enabling the evaluation of the objective function Eq. 1 in a significantly less computationally intensive manner than running a simulation, albeit with slightly reduced precision.

ANNs are computing systems inspired by biological neural networks found in animal brains (for a detailed description of ANNs, we refer to [29, Chapter 11] and [38, Chapter 2]). These systems can effectively capture complex interactions among inputs using hidden neurons. The neurons are units of computation that receive input from other neurons, perform computations on these inputs (such as performing a weighted sum before applying a nonlinear function commonly known as an activation function), and transmit their output to other neurons. If the ANN is structured as a Multi-Layer Perceptron (MLP), where neurons are arranged in layers with connections between all neurons of adjacent layers, it is well known that the ANN possesses universal approximation properties [20, 55]. These properties hold for certain choices of activation functions and a sufficient number of hidden neurons [42, 43].

Our motivation for employing an ANN-based metamodeling approach lies in the fact that sufficiently precise machine learning models, capable of accurately capturing the essential relationships between input and output variables, can provide efficient alternatives to computationally expensive DES models. Since the ANN metamodel provides an analytic expression of the objective function, our approach enables the solution of SBO problems using gradient-based methods. The complexity of the ANN metamodel depends on factors such as the number of hidden layers, the number of neurons, and the types of activation functions in the underlying MLP. Regardless of the metamodel’s complexity, the gradient of the MLP can always be computed.

We now describe the ANN-based metamodeling approach proposed to address the MOSBO problem in Eq. 7. This approach involves the following sequential steps:

1. *generation of the dataset* used to train the MLP;
2. *identification of suitable hyperparameters* for the training process;
3. *training* of the MLP;
4. *generation of Pareto optimal points* for the MOSBO problem when using the MLP to evaluate the objective function $f_1$ in Eq. 1.

In the following subsections, we provide a detailed description of these steps.

### Generation of the dataset

To train the ANN metamodel under the supervised learning paradigm, a dataset with the following structure is needed: $\{\textbf{v}_j, w_j\}_{j=1}^p$, where $\textbf{v}_j$ represents the input vector and $w_j$ represents a scalar target variable. For the specific application we are considering, for a given period $D=\{1, \ldots , n\}$, each $\textbf{v}_j$ corresponds to an ED setting $(x_1, \ldots , x_n, y_1, \ldots , y_n, r_1, \ldots ,$ $r_n, z)^\top$ (see Section 3.1).

After generating *p* random vectors $\textbf{v}_1 , \ldots , \textbf{v}_p$ satisfying constraints Eqs. 4, 5, and 6, the associated target values $w_1, \ldots ,w_p$ required to train the MLP underlying the ANN metamodel are obtained by running the DES model. Specifically, we compute the overall patient DTDT for each ED setting $\textbf{v}_j$ from the simulation output and store its value in the corresponding scalar $w_j$.

It is worth highlighting that this method of generating the dataset avoids many common issues encountered in datasets typically used to train machine learning models, such as missing values, outliers, and variations in the probability distribution of each data sample. Using runs of the DES model to generate data ensures high-quality data, allowing for control over the dataset size (adjustable based on the desired ANN metamodel accuracy) and facilitating the training of a reliable ANN metamodel. Given the high degree of nonlinearity and complex relationships between the vectors $\textbf{v}_j$ and the target variables $w_j$, an MLP with a sufficiently large number of hidden layers and neurons is typically identified as a suitable ANN architecture for learning this relationship [38]. It is important to note that the procedure described for generating the dataset is the only time-consuming phase of the approach we propose.

### Identification of the hyperparameters

Before training the MLP, it is necessary to decide the values of the hyperparameters involved in the training process. These hyperparameters include parameters that define the network’s architecture (such as the number of layers, number of neurons in each layer, and dropout rate) as well as parameters influencing the training process itself (such as those related to the optimization algorithm, number of epochs, and batch size). Due to the critical role of hyperparameters in determining the MLP performance, and for an overview of various selection strategies, we refer interested readers to [14].

To select an optimal configuration of hyperparameters, we implemented a random search with a *K*-fold Cross-Validation, a widely-used resampling procedure for determining hyperparameter values without biasing the assessment of the generalization capabilities of an ANN [11]. The *K*-fold Cross-Validation procedure works as follows: first, the training set is divided into *K* groups or folds. Then, *K* iterations are performed, where at each iteration *k* (with $k\in \{1, \ldots , K\}$), $K-1$ folds are used as the training set, while the remaining fold serves as the validation set. During each iteration, the MLP is trained using a specific configuration of hyperparameters, and an evaluation metric (such as the mean absolute error) is calculated to assess the MLP performance on the validation set. After completing all *K* iterations, the average value of the evaluation metric across the *K* different validation sets is computed and recorded. Finally, the configuration of hyperparameters yielding the minimum average value of the chosen metric is selected as the optimal configuration.

### Training of the MLP

Training a machine learning model involves adjusting its parameters to minimize the approximation error by reducing the discrepancy between the model’s predicted values and the actual values. This training procedure is typically achieved through the solution of an optimization problem, often employing the Stochastic Gradient Descent algorithm [12]. Once the training is complete, the MLP serves as a metamodel to compute approximate values of the function $f_1$ in Eq. 1, which is used in the MOSBO problem stated in Eq. 7.

### Generation of the Pareto optimal points

The solution of the multiobjective problem Eq. 7 is provided in terms of a Pareto front, which is a set of solutions that represent trade-offs between conflicting objectives. We obtain Pareto optimal points by using the well known *weighting method* (see, e.g., [61]). This method involves solving the following single-objective problem

9 $$\begin{aligned} \begin{array}{rl} \text {min} &{} \displaystyle \sum _{i=1}^3 \eta _i f_i(\textbf{x},\textbf{y},\textbf{r},z) \\ \text {s.t.} &{} {(\textbf{x}, \textbf{y}, \textbf{r}, z)\in \mathcal{F}}, \end{array} \end{aligned}$$

for different values of the weights $\eta _i$, with $\eta _i\ge 0$, $i=1, \ldots , 3$, and $\eta _1+\eta _2+\eta _3=1$. A solution to problem Eq. 9 serves as a (weak) Pareto optimal point (see [61]). While it may not determine all Pareto optimal solutions, in general, this method enables the construction of a good approximate Pareto front for a multiobjective optimization problem, and this explains its widespread usage.

When the function $f_1$ in Eq. 1 is replaced by its approximation obtained via the ANN metamodel, several significant advantages emerge:

- Evaluation of the objective function $f_1$ becomes extremely fast.
- Since the derivatives of $f_1$ can be evaluated by applying the backpropagation algorithm [66], all problem functions now have derivatives that can be numerically computed, allowing for the use of gradient-based optimization methods and leading to substantial efficiency improvements.
- Parallel or distributed computing techniques can be used to expedite the dataset generation phase by generating the dataset through concurrent processes, separate from the optimization process. Additionally, concurrent processes can be used to perform simultaneous minimizations, each corresponding to a combination of weights in problem Eq. 9.
- Additional objective functions and constraints that do not involve KPIs computed via the simulation model can be easily incorporated into the initial problem formulation, without the need to re-train the ANN metamodel.

Specifically, in scenarios where new constraints are introduced or terms in the objective functions are modified, the ANN metamodel exhibits its inherent adaptability by obviating the need to rerun the computationally demanding DES model. This adaptability arises from the surrogate nature of the pre-trained ANN metamodel, which allows for immediate rerunning of the optimization phase without additional training. Compared to the conventional approach of using the DES model, this strategy significantly accelerates the optimization process. The evaluation of the objective function through the trained ANN metamodel is accomplished in a fraction of the time typically required for a single DES model simulation. Moreover, since a single-objective minimization can be performed quickly, a great number of such minimizations can be carried out, leading to the generation of several points in the approximate Pareto front.

Note that an additional (optional) final step can be applied to the results of the optimization procedure. The value of the objective function $f_1$ corresponding to each Pareto optimal point, obtained using the ANN metamodel, is actually an approximation of the expected value of the overall patient DTDT. To enhance the accuracy of this value, each obtained point can be given as input to the DES model, and the actual DTDT can be computed via a simulation run. Of course, this requires additional computational time. In the case study we describe below, we adopt this strategy to improve the accuracy of the results.

## The case study

To test the proposed ANN-based metamodeling approach, we examine a real case study: the ED of a large Italian hospital, specifically, the ED of *Policlinico Umberto I* in Rome, Italy, which is significantly affected by the overcrowding problem. This ED is among the largest in Europe, with approximately 140,000 patient arrivals per year, and is composed of several areas addressing medical specialties that range from ophthalmology and hematology to obstetrics, pediatrics, and dentistry. In this paper, we focus on the *central area*, which provides treatments to patients whose conditions fall within the realms of internal medicine and general surgery. The empirical data used in our computational experiments was collected in 2018, when more than 50,000 patients used the emergency services offered in the central area of this ED. Note that the triage system in place in 2018 employed a four-level color-coded scale, assigning tags of *red*, *yellow*, *green*, and *white* in descending order of severity.^1^

**Fig. 1:**
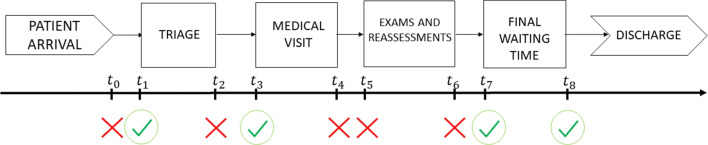
Scheme of ED patient flow and related timestamps. A green tick indicates that a timestamp is available from data

Some important aspects of this case study have been already deeply analyzed in the papers [22] and [23]. In particular, [22] examines the patient arrivals process in depth to determine the optimal piecewise constant approximation for the nonhomogeneous Poisson process arrival rate. Paper [23] addresses the problem of missing data, which affects many processes within the ED, by proposing a model calibration procedure to obtain a DES model that produces accurate output. We refer to these two papers for a detailed description of the ED operation. In the following sections, for the sake of completeness, we briefly describe aspects that are more relevant to the MIU optimal resource allocation problem we are addressing.

### The ED units

Two main units are used to visit and treat patients from all triage categories: a *Medical Unit* (MU), which specializes in diseases and disorders related to internal medicine, and a *Surgical Unit* (SU), which provides treatments to patients needing a surgical operation. Within these units, fully-equipped areas, denoted as MU_Red_ and SU_Red_, are dedicated to red-tagged patients. Moreover, a *Resuscitation Area* (RA) is available for cases requiring increased attention due to life-threatening diseases. In contrast, yellow-tagged and green-tagged patients do not have dedicated rooms and share resources within the MU and SU. Finally, a *Minor Injuries Unit* (MIU) is devoted to the visit and treatment of less urgent patients, including the least critical green-tagged patients and all white-tagged patients directed to the MIU by the triage nurse via the corresponding FT. Typically, the MIU operates Monday through Saturday from 7:00 a.m. to 8:00 p.m., with green tags assigned in place of white ones when the MIU is closed. All other units are open 24 hours a day, 7 days a week.

Regarding the number of rooms used for the medical visit and treatment of red-tagged patients, the RA always has 2 rooms available, while the MU and SU have 1 and 2 rooms, respectively. For the medical visit and treatment of yellow-tagged, green-tagged, and white-tagged patients, the MU and SU utilize 3 and 2 rooms during the daytime (8:00 a.m. to 8:00 p.m.) and 2 and 1 rooms during the nighttime (8:00 p.m. to 8:00 a.m.), respectively. Typically, 2 rooms are used in the MIU. In our study, although we report data related to the RA, we do not consider this unit in the experimentation since it is reserved exclusively for severe red-tagged patients with the highest priority, thereby avoiding any waiting time.

### The patient flow

Several diagnostic and therapeutic pathways can be identified within the ED based on the severity tag assigned to patients. In Fig. 1 we present a simplified scheme of the patient flow, highlighting the major blocks.

Specifically, in the examined ED, red-tagged patients can be directed to the MU_Red_ or SU_Red_ based on the required medical specialty, or to the RA in cases of critical health conditions. Yellow-tagged patients are only visited and treated in the MU and SU, where resources are shared with green-tagged patients not sent to the MIU. This latter decision, made by the triage nurse, depends on both whether the MIU is open or closed and the severity of the patient’s condition. In the MIU, green-tagged patients share available resources with white-tagged ones. Following medical visits and treatment, the subsequent stage includes exams (CT scans, X-rays, etc.) and reassessments. Some patients may also require observation periods between exams or before discharge. Again, a comprehensive description of all clinical pathways adopted in this ED can be found in [23].

### Data collection

Data collection is crucial for developing an accurate ED model. For the purpose of this study, we consider the data collected in January, since this month is considered particularly critical in terms of overcrowding level by the ED managers (of course, different months or time periods can be easily considered). In January 2018, 4192 patients arrived at the central area of the ED of Policlinico Umberto I. Table 1 reports the distribution of patients among severity tags and ED units assigned, excluding LWBS and deceased patients, as well as those directed to the orthopedic unit, which is not considered in this study since it concerns a different dedicated pathway.

**Table 1:** Distribution of patients among ED units and color tags

	White	Green	Yellow	Red	
MU	−	248 (21.83 %)	1316 (65.51 %)	191 (75.79 %)	1755
SU	−	628 (55.28 %)	693 (34.49 %)	45 (17.86 %)	1366
RA	−	−	−	16 (6.35 %)	16
MIU	47 (100 %)	260 (22.89%)	−	−	307
	47	1136	2009	252	

The table clearly shows that the majority of patients are yellow-tagged and that most of them are directed to the MU, while green-tagged patients are mostly sent to the SU. Most red-tagged patients require visits and treatment in the MU.

While data concerning tags assigned and resources scheduled is typically sufficiently complete (information such as assigned tags, physician and nurse shifts, and rooms available is usually recorded), there is often a lack of detailed data on the timestamps for each patient activity within an ED. This is a well known problem in the literature on EDs (see [23] and the references reported therein). Regarding our case study, Fig. 1 reports the timestamps of the main processes in the patient flow, highlighting those available and unavailable. For a detailed description of all the timestamps and the reasons for the unavailability of some of them, we again refer to [23]. We just recall here that since timestamps $t_2$, $t_4$, $t_5$, and $t_6$ are unavailable, we cannot directly compute the service times of triage, medical visits, and examinations and reassessments from the data, and we use the procedure described in [23] to estimate these service times.

### The discrete event simulation model

A DES model of the patient flow within the considered ED was implemented using the R package Simmer [74], which allows building process-oriented DES models based on trajectories. For a complete description of the DES model of the ED under study, once again, we refer to [23]. Here we simply recall that the model entities are patients who enter the model according to a proper arrival process, namely, a piecewise constant approximation of a nonhomogeneous Poisson process (see [22] for a detailed description of this process). After triage, entities follow different trajectories according to the received severity tag (which is stored as an attribute) and the assigned ED unit. The combination of the color tag and the ED unit determines the different patient flows. The resources used in the model represent the rooms for medical visits and treatments. While the rooms of the MU_Red_, SU_Red_ and RA have fixed capacities, the rooms of the MU, SU, and MIU are based on a schedule that reflects the change in capacity between day and night shifts.

For the model validation and the design of experiments, we refer to [23] and adopt the same choices here, i.e., a simulation length of 38 days, a warm-up period of 1 week, and a fixed number of replications set to 30.

## Statement of the MIU optimal resource allocation problem for the case study

The simulation model of the ED of Policlinico Umberto I is used to achieve the final goal of reducing the level of overcrowding. Through interviews with the ED managers, a significant interest emerged in exploring the possibility of accommodating a larger number of green-tagged patients in the MIU. This interest arose because triage nurses, responsible for assigning color tags, often adopt a cautious approach, directing fewer patients to the MIU than the ones it can accommodate. They do so out of concern for potential liability if a patient’s condition worsens due to a misjudged ED unit assignment at triage. However, reducing overcrowding by balancing the workload across ED units requires careful monitoring of the overall number of patients to prevent the benefits achieved in one unit from leading to long waiting times in other units of the ED.

Naturally, diverting more patients to the MIU is just one potential strategy to decrease overcrowding. Another tool considered by the ED managers is adjusting the working hours of the MIU, which currently operates from 8:00 a.m. to 8:00 p.m., Monday through Saturday. Given the high flexibility of the ED staff, managers consider any combination of MIU opening and closing times throughout the week feasible, provided it leads to an improvement over the current situation and falls within the 7:00 a.m. to 8:00 p.m. timeframe. Of course, the largest reduction in waiting times is expected for low-acuity patients, i.e., green-tagged or white-tagged patients, since these are the categories assigned to the MIU. As observed in [69], more critical patients may experience a lower reduction in waiting times due to their priority, which guarantees shorter waiting times. In particular, some benefits are expected for yellow-tagged patients, who will share resources with a lower number of low-acuity patients. In contrast, red-tagged patients rely on dedicated resources, and accordingly, no improvement is expected for them.

Let us now formally state the MIU optimal resource allocation problem. By using the notation introduced in Section 3, we define ${\mathcal {T}}=\{R, Y, G, W\}$ as the set of severity (color) tags: red (*R*), yellow (*Y*), green (*G*), and white (*W*). Moreover, for each $t\in \mathcal{T}$, we have $U(t) \subseteq \{\textrm{MU}, \textrm{SU}, \textrm{RA}, \textrm{MIU}\}$, where

$$\begin{aligned} U(t) = {\left\{ \begin{array}{ll} \{\textrm{MIU}\} \qquad \qquad \qquad \text { if } \quad t=W, \\ \{\textrm{MU, SU, MIU}\} \quad \text { if } \quad t=G, \\ \{\textrm{MU, SU}\} \qquad \qquad \text { if } \quad t=Y, \\ \{\textrm{MU, SU, RA}\} \ \ \quad \text { if } \quad t=R. \end{array}\right. } \end{aligned}$$

As we mentioned at the end of Section 6.1, we do not consider the RA in our experimentation, which implies $U(R)=\{MU,SU\}$. Since a weekly planning is adopted, we have $D=\{1, \ldots , 7\}$. The decision variables include the opening and closing times for each weekday, denoted as $x_d$ and $y_d$, with $d\in D$, as well as the percentage of patients diverted to the MIU via the FT system. The need to differentiate this percentage between morning and afternoon emerged from observing the data and conducting interviews with ED personnel. Therefore, we introduce two variables, $z_1$ and $z_2$, to denote these percentages. The upper bounds for these variables are directly deduced from real data by observing the maximum values of these percentages in the morning and afternoon for each day of the week. For this case study, the variables $r_d$ and the objective function $f_3$ in Eq. 3 are omitted from our problem formulation because the number of rooms used in the MIU of the examined ED is fixed.

The resulting MOSBO problem can then be formulated as follows:

10 $$\begin{aligned} \begin{array}{rl} \text {min} &{} \left( f_1(\textbf{x},\textbf{y},z_1,z_2) , f_2(\textbf{x},\textbf{y}) \right) ^\top \\ \text {s.t.} &{} {(\textbf{x}, \textbf{y}, z_1, z_2)\in \mathcal{F}}, \end{array} \end{aligned}$$

where the feasible set ${\mathcal {F}}$ is defined by constraints Eqs. 4–6, and the functions $f_1$ and $f_2$ are defined in Eqs. 1 and 2, respectively. This problem constitutes a bi-objective mixed integer SBO problem, involving 15 variables and 22 constraints. We employ the sample average approximation method, as described in Section 3.4, to approximate the expected value in function $f_1$. In particular, runs of the ED DES model described in Section 6.4 are used to evaluate the sample average response functions associated with the overall patient DTDT. Here, the DTDT for each patient can be computed from the difference $t_3-t_1$, where $t_1$ and $t_3$ are the timestamps in Fig. 1. Note that we approximate the DTDT using $t_3-t_1$ (i.e., the time difference between the start of triage and the start of the medical visit) instead of $t_3-t_0$ because the arrival time $t_0$ is unavailable from the data. This approximation amounts to assuming that the triage begins when a patient arrives in the ED (i.e., $t_0=t_1$).

The weighting method applied to problem Eq. 10 for determining Pareto optimal points requires solving the following single objective problem

11 $$\begin{aligned} \begin{array}{rl} \text {min} &{} \displaystyle \eta _1 f_1(\textbf{x},\textbf{y},z_1,z_2)+\eta _2 f_2(\textbf{x},\textbf{y},z_1,z_2) \\ \text {s.t.} &{} {(\textbf{x}, \textbf{y},z _1, z_2)\in \mathcal{F}}, \end{array} \end{aligned}$$

for different values of $\eta _1\ge 0$ and $\eta _2\ge 0$, where $\eta _1+\eta _2=1$.

## Experimentation

In this section, we present the results of computational experiments performed to solve the MIU optimal resource allocation problem for the case study described in Section 6. In this experimentation, we consider problem Eq. 10 with the following specifications: the function $f_1$ in Eq. 1 with $\alpha _t=1$, for all $t\in \mathcal{T}$ and $\beta _u=1$ for all $u\in U(t)$, and the function $f_2$ in Eq. 2 with $\gamma _d=1$, for $d=1, \ldots , 7$. These weight values are chosen to assign equal importance to all terms in the respective functions. Regarding the problem constraints, we set lower and upper bounds on the variables $x_d$ and $y_d$ in the box constraints Eq. 4 to 7:00 a.m. and 8:00 p.m., respectively (i.e., $l_{x_d}=l_{y_d}=7$ and $u_{x_d}=u_{y_d}=20$ for $d=1, \ldots ,7$). For the variables $z_1$ and $z_2$, according to indications of the ED personnel, we set $l_{z_1}=l_{z_2}=0$ and $u_{z_1}=75$, $u_{z_2}=35$. In constraints Eq. 5, we set $h_d= 0$ for $d=1, \ldots , 7$ to allow for the possible closure of the MIU on certain days of the week. In Eq. 6, as suggested by the ED managers, we choose $g=21$ to ensure a minimum number of MIU open hours during the week.

All the tests were performed on a PC equipped with an Intel Core i7-4790K Quad-Core 4.00 GHz Processor with 32 GB RAM. For the implementation of the ANN metamodel, we used Python 3.8.10. In particular, we implemented the MLP using the PyTorch library. The MLP hyperparameters were determined through a random search with $K=5$ in the *K*-fold Cross Validation, with the mean absolute error employed as a metric. The training was performed by using ADAM optimizer [48] with a learning rate set to $10^{-5}$. We used 574 epochs with a batch size of 4 and an early stopping patience set to 8 epochs. The resulting network is composed of two hidden layers, each containing 90 neurons. We employed the Rectified Linear Unit (ReLU) as the activation function.

The starting point of each single objective minimization of the “scalarized" problem Eq. 9 is given by $(\textbf{x}^0, \textbf{y}^0, z^0_1, z^0_2)$, which corresponds to the “as-is” status, i.e., the current MIU opening and closing times ($\textbf{x}^0$ and $\textbf{y}^0$, respectively) and the current average percentages of patients directed to the MIU by the triage nurse in the morning and afternoon ($z_1^0$ and $z_1^0$, respectively). The values of each component of this starting point are reported in Table 2 (note that the MIU is currently closed on Sundays) along with the corresponding values $f_1^0=f_1(\textbf{x}^0, \textbf{y}^0, z^0_1, z^0_2)$ (in minutes) and $f_2^0=f_2(\textbf{x}^0, \textbf{y}^0, z^0_1, z^0_2)$ (in hours), which are provided for reference.

**Table 2:** Starting point of the optimization algorithm associated with the “as-is” status and corresponding values $f_1^0$ (in minutes) and $f_2^0$ (in hours)

$x_1^0$	$y_1^0$	$x_2^0$	$y_2^0$	$x_3^0$	$y_3^0$	$x_4^0$	$y_4^0$	$x_5^0$	$y_5^0$	$x_6^0$	$y_6^0$	$x_7^0$	$y_7^0$	$z_1^0$	$z_2^0$	$f_1^0$	$f_2^0$
8	20	8	20	8	20	8	20	8	20	8	20	8	8	45	5	734.34	72

**Table 3:** Pareto optimal solutions and corresponding values $f_1$ (in minutes) and $f_2$ (in hours)

	$x_1$	$y_1$	$x_2$	$y_2$	$x_3$	$y_3$	$x_4$	$y_4$	$x_5$	$y_5$	$x_6$	$y_6$	$x_7$	$y_7$	$z_1$	$z_2$	$f_1$	$f_2$
***A***	7	18	9	20	9	20	7	19	7	20	10	20	7	19	65	35	701.23	80
***B***	8	19	10	20	9	19	7	18	8	19	10	20	7	18	70	35	702.52	74
***C***	9	20	10	20	7	16	7	18	9	20	9	17	7	15	65	35	705.23	68
***D***	8	19	10	20	7	16	7	18	9	20	8	16	7	13	60	35	708.98	66
***E***	10	20	11	20	7	15	10	18	9	20	9	14	7	12	60	35	711.09	56
***F***	10	20	10	19	8	16	10	18	9	20	9	13	7	12	60	35	714.55	55
***G***	12	20	9	17	9	17	10	15	9	20	10	14	7	11	65	35	719.00	48
***H***	12	20	9	16	10	17	10	15	9	20	10	14	7	11	65	35	722.95	46
***I***	12	20	9	16	10	17	11	14	9	20	10	14	7	10	65	35	733.92	43
***J***	12	20	8	14	10	16	11	13	9	19	9	13	7	7	65	35	736.74	36
***K***	7	7	10	13	11	16	11	14	9	16	12	15	7	7	60	35	766.11	21

We set the maximum overall number of runs of the DES model to 10, 000 (we recall that each run consists of 30 independent replications). The ANN-based metamodeling approach allocates this entire budget of runs to the dataset generation phase. As mentioned previously in Section 4.2, this phase represents the most time-consuming aspect of the proposed metamodeling approach. After completing the training of the ANN metamodel on the generated dataset, the time required to evaluate the function $f_1$ using the ANN metamodel becomes negligible. Therefore, we can afford to conduct a large number of single objective minimizations for problem Eq. 11, each corresponding to different combinations of the weights $\eta _1$ and $\eta _2$. This approach allows us to generate several Pareto optimal solutions and obtain a good coverage of the Pareto front. In particular, we consider 101 combinations of the weights $(\eta _1, \eta _2)$: $\{(1,0), ~ (0.99, 0.01), ~ \ldots ~ , (0.01 , 0.99) , ~ (0,1)\}$. We note that weights with values close to each other may lead to the same minimizer in different single objective minimizations. For the single objective minimizations, we employ a standard SQP algorithm for constrained nonlinear optimization available in the Scipy library. Integer variables are treated as continuous variables, and their values are then rounded to the nearest integers.

In Table 3, we present the distinct non-dominated Pareto optimal points along with the corresponding values for functions $f_1$ and $f_2$.

**Fig. 2:**
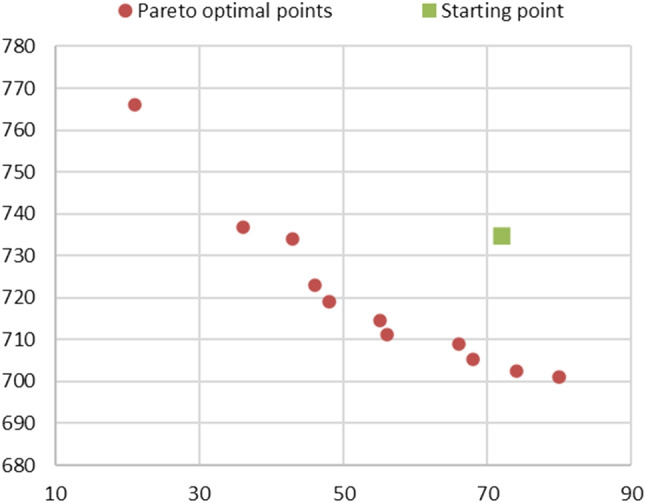
Pareto optimal solutions obtained using the ANN-based metamodeling approach are depicted in the objective space, with $f_1$ (in minutes) represented on the ordinate axis and $f_2$ (in hours) on the abscissa axis. The green square represents the point corresponding to the current “as-is” status, which serves as the starting point of the optimization procedure

In Fig. 2, the resulting approximate Pareto front is depicted in the objective space (i.e., the space where the objective values are used as coordinates). The point corresponding to the starting point is also included.

From Fig. 2, we can observe that the obtained Pareto optimal points provide a good coverage of the Pareto front. However, the distribution of points is more concentrated for values of $f_2$ (abscissa axis) exceeding 30 hours. This is due to the limited number of MIU opening hours during the week, resulting in fewer Pareto optimal solutions. We also highlight that the starting point corresponding to the “as-is” status is dominated by the generated Pareto optimal points, indicating that the current status is suboptimal based on the selected criteria. The extreme points ***A*** and ***K*** result from separate minimizations of $f_1$ and $f_2$, respectively. These extreme points are likely unrealistic: ***A*** entails excessively high costs, while in the ED setting corresponding to ***K***, patient waiting times (measured by the objective function $f_1$) are significantly high. In contrast, the intermediate points represent trade-off solutions, offering ED managers various options to choose from while ensuring Pareto optimality.

Observing the ED settings corresponding to the Pareto optimal points in Table 3 yields several interesting conclusions. Firstly, there is a noticeable increase in the percentage of patients directed to the MIU via the FT compared to the “as-is” status. This increase is relatively modest in the morning, rising from 45% to 60-70%, but more pronounced in the afternoon, varying from 5% to 35%. This suggests the need for ED managers to explore enhanced triage methods to assess the feasibility of directing a greater number of patients to the MIU via the FT, particularly during afternoon hours. Additionally, except for settings ***J*** and ***K***, MIU is expected to be operational on Sundays, whereas it is currently closed. Moreover, in many cases, the opening and closing times deviate from the ones currently adopted (8:00 a.m. to 8:00 p.m.), with some solutions proposing earlier or later opening times and earlier closing times.

It is important to note that, although in most cases the MIU is open for fewer hours compared to the current “as-is” status, the optimized distribution of opening hours throughout the week facilitates the redirection of more patients to the MIU while minimizing waiting times. The primary source of this improvement lies in the adoption of an optimal resource allocation strategy, allowing for the customization of MIU opening hours based on actual requirements. This clearly shows the necessity for greater flexibility in determining the MIU operational schedule.

For brevity, we do not report the patient DTDT for each color tag and ED unit. However, it is worth noting that the findings confirm our expectations: patient waiting times for red-tagged and yellow-tagged patients remain largely unchanged due to their high priority. Conversely, improvements are observed for green-tagged and white-tagged patients, who are directly impacted by potential diversion to the MIU via the FT.

### Comparison with the use of Derivative-Free Optimization

The aim of this section is to compare the proposed ANN-based metamodeling approach with a DFO-based approach, which consists of applying a DFO algorithm to solve the considered SBO problem without using a metamodel. Specifically, in the DFO approach, we again solve the bi-objective problem Eq. 10 using the weighting method, i.e., solving a series of single objective optimization problems Eq. 11 for several weight combinations. However, we now evaluate the objective function $f_1$ directly by running the DES model and using the sample average approximation method described in Section 4.1. In other words, we employ the standard simulation optimization approach: during each iteration of the DFO algorithm, the trial point (representing an ED setting) is passed to the DES model, and the resulting output is used to approximate each expected DTDT in the objective function $f_1$ in Eq. 1.

For the DFO approach, we decided to adopt the DFLINT algorithm, which is an effective DFO algorithm recently proposed in [59]. This algorithm employs a novel strategy for solving black-box problems with integer variables, using well-suited search directions and a non-monotone linesearch procedure. Even in cases of early termination, needed for limiting the computational burden, it still produces points that are guaranteed to approximate local minimum points. Moreover, the accuracy of the obtained solution can be easily controlled by means of the stopping criterion. This algorithm is available with GNU GPL license on the web^2^. Note that in some healthcare problem instances, algorithms belonging to the class of linesearch-based DFO methods have been proven to be much more efficient than optimization engines commonly used in the area of simulation optimization (see [60] for a comparison with OptQuest).

In the experiments, we use the default parameters of the DFLINT algorithm, with the stopping criterion based on the maximum number of function evaluations. To ensure a fair comparison between the ANN-based metamodeling approach and the DFO approach, we allocate the same overall budget of 10,000 function evaluations. In the ANN-based metamodeling approach, these evaluations are used as the maximum overall number of runs of the DES model for generating the dataset. In the DFO approach, these evaluations are equally divided into 11 single-objective minimizations of problem Eq. 11, each corresponding to one of the following combinations of weights: $\{(1,0), ~ (0.9, 0.1), ~(0.8, 0.2) , ~ \ldots ~ , (0.1 , 0.9) , ~ (0,1)\}$. In particular, for each single objective minimization, the DFLINT algorithm is subject to a stopping criterion of 909 function evaluations (where 909 results from the division 10,000/11). The rationale behind partitioning the budget in this manner is based on the fact that allocating a larger number of single objective minimizations would result in a very small number of function evaluations available for each, reducing the accuracy of the solutions obtained from each minimization. The same starting point considered previously and given in Table 2 is used.

**Fig. 3:**
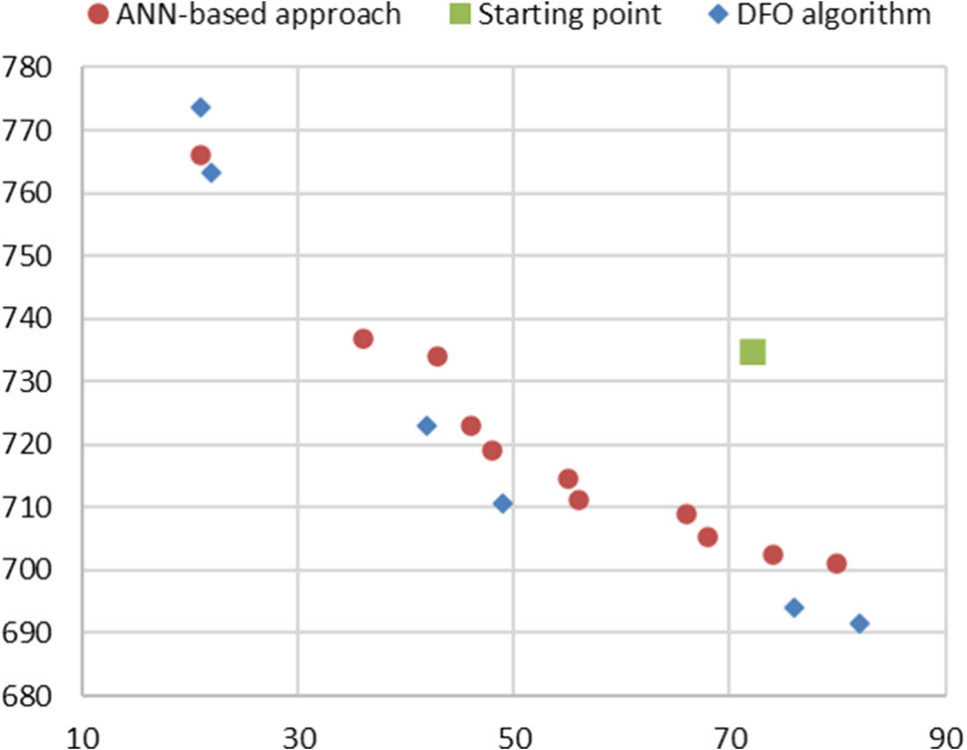
Pareto optimal solutions obtained by the ANN-based metamodeling approach (red bullets) and the DFO algorithm (blue diamonds). The green square corresponds to the starting point

In Fig. 3, we present a comparison between the Pareto optimal points obtained by the ANN-based metamodeling approach (red bullets) with those obtained by the DFO approach (blue diamonds), without providing their values for the sake of brevity. Figure 3 clearly evidences that the ANN-based approach provides us with a wider range of trade-off solutions. In fact, a greater number of Pareto optimal solutions are generated, leading to a better coverage of the Pareto front. In practical terms, the ANN-based approach offers decision-makers a more extensive array of options to consider, which represents a significant advantage of our proposed method. Conversely, the application of a DFO algorithm in the DFO approach demands considerable computational resources, since each function evaluation necessitates runs of the DES model. This computational burden limits the exploration of a large number of weight combinations when employing the weighting method. The small coverage of the Pareto front is the major drawback of using the DFO approach.

Figure 3 also highlights that the DFO algorithm tends to generate solution points that dominate the ones obtained by the ANN-based approach, even if the difference in the corresponding function values is very small. This is expected because greater accuracy can be achieved by employing an exact algorithm like the one adopted in the DFO approach. We also believe that the use of more sophisticated techniques, such as *active learning* (see, e.g., [70]) could significantly improve the proposed ANN-based approach, leading to overall greater accuracy.

### Results from sensitivity analysis experiments

The results of the experimentation we reported are obtained by setting $\alpha _t=1$ for all $t\in \mathcal{T}$ and $\beta _u=1$ for all $u\in U(t)$ in the definition of the function $f_1$ in Eq. 1, and $\gamma _d=1$ for $d=1, \ldots , 7$ in the definition of the function $f_2$ in Eq. 2. The rationale behind this choice is to assign equal importance to all terms in these objective functions. However, it is worth noting that different values for these coefficients could be chosen to prioritize certain terms over others. To evaluate the impact of such variations, we conducted a sensitivity analysis. It is important to note that the proposed ANN-based metamodeling approach facilitates the execution of sensitivity analyses with minimal computational effort. Without using this approach, conducting such analyses through the combined use of a computationally expensive DES model with a DFO algorithm would be highly costly, thereby limiting the scope of feasible experimentation for this analysis. We provide a summarized overview of the extensive analysis we performed below.

Regarding the coefficients $\alpha _t$, $t\in {\mathcal {T}}$, associated with patient tags, we expect minimal effects when varying their values. This is because the priority-based mechanism of the triage tags governs patient flow. Tests performed by varying the coefficients $\alpha _t$ confirmed our expectation: even when assigning greater values to coefficients associated with less severe tags (such as green and white), only slight modifications were observed in the resulting waiting times. As expected, waiting times for more severe tags remained relatively shorter. Given the limited significance of the analysis in this case, detailed results are not provided.

A greater sensitivity is observed when varying coefficients $\beta _u$, $u\in U(t)$, associated with the ED units. We adopt two different representative choices of $\beta _u$, $u\in U(t)$, as reported in Table 4, setting $\gamma _d=1$ for all $d\in D$ and $\alpha _t=1$ for all $t\in \mathcal{T}$. In Tables 6 and 7 in Appendix A, detailed results are presented. Alongside the optimal values of variables and functions ($f_1$, $f_2$), these tables provide the actual (unweighted) values of the first objective function, denoted by ${\hat{f}}_1$. Additionally, they include the aggregated patient waiting times for each ED unit, regardless of the triage tags of the patients directed to these units. Comparing these tables, it can be observed that adopting a higher value, such as $\beta _{\text {MIU}} = 0.6$ (choice *(i)* in Table 4), leads to a greater allocation of opening hours for the MIU. Conversely, choosing $\beta _{\text {MIU}} = 0.2$ (choice *(ii)* in Table 4) results in a reduction of the MIU opening hours, aligning with expectations. To offer a comprehensive understanding of the impact on waiting times across all ED units, average values of the aggregated waiting times for each unit are included in Table 5. These values are computed by averaging the aggregated waiting times over all obtained Pareto optimal points reported in Tables 6 and 7.

**Table 4:** Two representative choices for $\beta _u$, $u\in U(t)$

	$\beta _\textrm{SU}$	$\beta _\textrm{MU}$	$\beta _\textrm{MIU}$
*(i)*	0.2	0.2	0.6
*(ii)*	0.5	0.3	0.2

It is evident that the aggregated waiting times decrease as the coefficients $\beta _u$ assigned to the corresponding ED units increase. In contrast, whenever a lower value of $\beta _u$ is used, increased aggregated waiting times are observed for the corresponding ED unit. The observed behavior in these two example choices is consistent with the results from all the other tests we conducted.

We now focus on analyzing the coefficients $\gamma _d$, $d\in D$, which assign different weights to each day of the week in the objective function $f_2$. For brevity, we present results for selected values of $\gamma _d$, while maintaining $\alpha _t=1$ for all $t\in \mathcal{T}$ and $\beta _u=1$ for all $u\in U(t)$. Specifically, we highlight the effects of adopting different coefficients by reporting detailed results for an extreme case where some coefficients are intentionally chosen to be very high. This case is represented as follows:

$$\begin{aligned} \gamma _d=7, \, d=1, \ldots , 5, ~ \text {and} ~ \gamma _6=\gamma _7=7000. \end{aligned}$$

**Table 5:** Cumulative waiting times (in minutes) for each ED unit corresponding to the choices *(i)* and *(ii)* for $\beta _u$, $u\in U(t)$, specified in Table 4

	SU	MU	MIU
*(i)*	257.88	347.61	129.50
*(ii)*	256.63	340.79	133.68

This choice consists of assigning uniform values to the coefficients $\gamma _d$ associated with weekdays (Monday to Friday), while a substantially higher value is assigned to Saturday and Sunday. Note that large values of the coefficients $\gamma _d$ are necessary to achieve the desired effect due to the different magnitudes of the two objective functions, $f_1$ and $f_2$.

In Table 8 of Appendix A, we provide detailed results. This table includes the optimal values of variables and functions ($f_1$, $f_2$), as well as the actual (unweighted) values of the second objective function, denoted by ${\hat{f}}_2$, representing the actual number of MIU opening hours during the week. Observing Table 8, as expected, all the obtained Pareto optimal points suggest closing the MIU on the weekend. Different distributions of coefficients $\gamma _d$ lead to expected results: typically, choosing high coefficients associated with specific days of the week implies a decrease in MIU opening hours (or even closure) on those days (hence we do not provide further detailed results). Experiments not included in this paper also demonstrated the possibility of suitably tuning coefficients $\gamma _d$ to achieve desired results on MIU opening hours.

While the sensitivity analysis presented in this section is not exhaustive, we have provided results for specific representative settings of the coefficients in the two objective functions. Our experimentation demonstrates the robustness and effectiveness of both the problem formulation and the ANN-based metamodeling approach proposed in our paper. Furthermore, the analysis highlights the flexibility offered by the coefficients $\alpha _t$, $\beta _u$, and $\gamma _d$, allowing decision-makers to tailor solutions to meet specific needs.

## Conclusions

We examined the use of an FT system for low-acuity patients, a strategy commonly adopted to alleviate ED overcrowding. In particular, we focused on an optimal resource allocation problem in the MIU, the ED unit where such patients are possibly diverted via the corresponding FT. We formulated this problem as a multiobjective optimization problem, aiming at minimizing conflicting objective functions. Some of these objectives are computationally expensive black-box functions, requiring time-consuming simulations for their evaluation. Therefore, the problem at hand is a multiobjective SBO problem that requires high computational effort when using the standard approach based on DFO algorithms. To efficiently solve this problem, we developed a metamodeling approach that uses an ANN to replace a black-box objective function with a suitable metamodel.

To show the reliability and effectiveness of the proposed ANN-based metamodeling approach, we considered a real case study, namely, the ED of a large hospital in Rome, where an FT for low-acuity patients is currently adopted. We formulated the related MIU optimal resource allocation problem as a bi-objective SBO problem. The aim was to minimize both the expected overall patient DTDT and the operating costs of the MIU. Leveraging the available data, we developed a detailed DES model to simulate patient flow in the ED. Since our experimentation confirmed the significant computational burden associated with computing the expected overall patient DTDT through runs of this simulation model, we replaced this function with an ANN metamodel. This approach facilitated the generation of multiple Pareto optimal solutions, providing decision-makers with valuable insights. Our experimental results further underscored the suboptimal nature of the current ED setting based on the selected criteria. A comparison with the results obtained using the standard DFO approach highlighted the strengths of our proposed methodology. While our experimental case study showed that the DFO approach tends to generate Pareto optimal points that dominate those obtained by the ANN-based metamodeling approach, the latter is generally preferred due to the following key advantages:

- The generation of a larger number of Pareto optimal solutions (compared to the DFO approach), which cover a wider range of trade-off solutions to be provided to ED managers.
- The possibility of using parallel or distributed computing techniques to expedite the dataset generation phase by running concurrent processes, enabling simultaneous executions of the DES model. This feature offers substantial time savings, particularly when compared to the sequential runs of the DES model required in the DFO approach.
- The adaptability to changes: the ANN-based metamodeling approach demonstrates robust resilience when new objective functions or constraints need to be incorporated within the problem formulation. If these adjustments do not affect the KPIs computed via the simulation model, the ANN metamodel can efficiently generate new Pareto optimal solutions. Notably, evaluating a single objective function value with our trained ANN metamodel requires mere hundredths of a second, substantially less than the thirty seconds to a minute needed with the DES model. This leads to a marked improvement in the computational speed of the optimization phase through the use of gradient-based optimization methods, which do not require additional costly simulation runs.

This efficiency is further illustrated by our sensitivity analysis (Section 8.2), which would have been impractical with the DES model alone due to the prohibitive computational demands.

On the other hand, our approach has some limitations. The first one concerns the specific formulation we adopted. To accommodate the requests of the ED managers in our case study, our approach assumes flexible MIU opening hours. Therefore, it is suitable for all EDs where MIU personnel can adjust their shifts to align with the chosen opening and closing times. Of course, if this flexibility is not suitable for a particular setting, a different problem formulation must be used. Another limitation lies in determining the values of the upper bound on the percentage of patients diverted to the MIU by the triage nurse. In general, this can be a complex task, depending on the specific dynamics of the ED. However, for EDs where the FT for low-acuity patients is already in place, conducting accurate data analysis can greatly assist in making an initial choice that reflects the current “as-is” status. If different scenarios lead to unrealistic results, different values of the upper bound on this percentage must be selected.

Future work will involve several aspects. From a modeling perspective, a potential avenue of research would be to minimize the maximum waiting time among all patients for each triage tag to ensure that no low-acuity patients wait significantly longer than others. Moreover, as already mentioned at the end of Section 8, one can study more sophisticated learning techniques to further improve result accuracy. Finally, one can explore specific algorithms, such as the one recently proposed in [73], to address the so called “heterogeneous” multiobjective optimization problems. In this context, “heterogeneous” refers to cases where one objective function is an expensive black-box function while others are given analytically. Note that even if the MOSBO problem stated in Eq. 7 falls into this class of problems, the algorithm proposed in [73] cannot be directly applied since it is designed for unconstrained problems, whereas the MOSBO problem involves both box and linear constraints.

## Data Availability

Data are available but, since they contain confidential information, they cannot be shared openly.

## A: Appendix

In this appendix we report detailed results of our experimentation concerning the sensitivity analysis considered in Section 8.2. In particular, Tables 6 and 7 report the results obtained for different values of the coefficients $\beta _u$ in the objective function $f_1$. Table 8 includes the results obtained for different values of coefficients $\gamma _d$ in the objective function $f_2$.

**Table 6:** Results obtained for $\beta _\textrm{SU}=0.2,$ $\beta _\textrm{MU}=0.2$, and $\beta _\textrm{MIU}=0.6$

$x_1$	$y_1$	$x_2$	$y_2$	$x_3$	$y_3$	$x_4$	$y_4$	$x_5$	$y_5$	$x_6$	$y_6$	$x_7$	$y_7$	$z_1$	$z_2$	$f_1$	$f_2$	${\hat{f}}_1$	WT_SU_	WT_MU_	WT_MIU_
7	20	7	20	7	20	7	20	7	18	7	20	7	20	50	35	578.16	89	697.90	254.74	310.31	132.85
7	20	7	20	7	20	7	17	7	17	7	20	7	20	45	30	578.59	85	702.55	254.39	317.28	130.88
7	20	7	15	7	20	7	20	7	19	7	20	8	20	40	25	582.08	84	706.68	253.07	321.88	131.73
7	20	7	20	7	20	7	15	7	19	13	19	7	20	45	35	585.62	78	712.72	257.22	323.84	131.66
7	20	15	20	7	20	7	14	7	20	7	11	7	19	40	35	585.96	67	719.86	257.32	334.17	128.37
7	18	7	20	8	20	7	15	11	20	7	14	20	20	40	35	587.70	60	719.28	255.50	333.67	130.11
11	16	7	20	10	20	13	18	7	18	15	20	10	20	50	35	594.45	59	731.64	257.40	344.68	129.56
7	12	7	20	11	20	12	20	7	16	20	20	16	20	50	35	599.80	48	743.02	258.33	356.36	128.32
12	14	20	20	7	18	7	14	7	20	7	16	10	13	30	35	604.15	45	749.45	259.65	361.07	128.73
14	20	7	14	17	20	7	12	12	20	7	13	13	20	30	35	618.65	42	772.89	261.79	382.01	129.09
7	12	8	18	14	20	19	20	7	14	15	19	16	20	40	35	618.91	37	778.01	263.58	387.68	126.75
7	12	20	20	17	20	13	14	13	16	14	18	9	14	65	35	622.92	21	786.13	261.69	398.40	126.04

The results include Pareto optimal solutions, objective function optimal values ($f_1$, $f_2$), actual (unweighted) values ${\hat{f}}_1$, and the corresponding values for the aggregated patient waiting times (WT) for each ED unit, regardless of the triage tags of the patients directed to these units

**Table 7:** Results obtained for $\beta _\textrm{SU}=0.5,$ $\beta _\textrm{MU}=0.3$, and $\beta _\textrm{MIU}=0.2$

$x_1$	$y_1$	$x_2$	$y_2$	$x_3$	$y_3$	$x_4$	$y_4$	$x_5$	$y_5$	$x_6$	$y_6$	$x_7$	$y_7$	$z_1$	$z_2$	$f_1$	$f_2$	${\hat{f}}_1$	WT_SU_	WT_MU_	WT_MIU_
11	20	9	20	7	16	10	19	10	20	8	14	8	16	60	35	742.96	62	701.55	252.84	314.91	133.80
11	20	9	20	7	16	10	19	10	20	8	14	9	16	60	35	746.48	61	704.14	254.85	315.44	133.85
11	20	10	20	8	16	10	18	10	20	8	14	7	14	60	35	750.30	58	710.06	253.15	321.46	135.46
11	20	10	20	8	16	10	18	10	20	8	13	7	14	60	35	754.72	57	712.87	255.22	324.34	133.32
9	20	8	14	7	16	10	15	9	19	8	15	7	7	7	35	759.42	48	718.00	256.81	324.98	136.21
11	20	9	15	8	16	10	14	10	20	9	17	7	7	70	35	766.99	45	723.60	259.90	329.73	133.97
11	20	12	17	9	17	10	15	9	20	10	13	7	7	65	35	774.30	41	734.73	254.57	347.82	132.34
10	18	9	16	7	17	13	15	8	18	12	15	7	7	60	35	783.29	40	741.50	258.87	351.36	131.27
12	20	14	16	8	14	8	15	10	20	8	14	7	7	65	35	790.23	39	751.70	256.35	361.65	133.70
12	20	15	18	8	14	9	15	10	20	10	13	7	7	65	35	793.50	36	752.56	259.90	360.19	132.47
12	13	12	17	11	16	9	13	10	15	9	11	13	13	70	35	796.83	22	755.81	259.67	365.48	130.67
11	13	12	15	12	17	11	15	9	14	10	12	12	12	65	35	799.56	21	760.33	257.45	372.19	130.68

The results include Pareto optimal solutions, objective function optimal values ($f_1$, $f_2$), actual (unweighted) values ${\hat{f}}_1$, and the corresponding values for the aggregated patient waiting times (WT) for each ED unit, regardless of the triage tags of the patients directed to these units

**Table 8:** Results obtained for $\gamma _1=\gamma _2=\gamma _3=\gamma _4=\gamma _5=7$ and $\gamma _6=\gamma _7=7000$

$x_1$	$y_1$	$x_2$	$y_2$	$x_3$	$y_3$	$x_4$	$y_4$	$x_5$	$y_5$	$x_6$	$y_6$	$x_7$	$y_7$	$z_1$	$z_2$	$f_1$	$f_2$	${\hat{f}}_2$
9	20	11	17	9	16	7	12	9	20	13	13	7	7	70	35	718.55	280	40
9	20	12	18	8	16	8	12	10	20	13	13	7	7	65	35	720.68	273	39
11	20	14	20	7	12	7	12	10	19	11	11	12	12	70	35	732.91	238	34
17	20	14	19	7	9	7	10	10	18	13	13	7	7	70	35	749.03	147	21

The results include Pareto optimal solutions, objective function optimal values ($f_1$, $f_2$) and actual (unweighted) values ${\hat{f}}_2$

## Footnotes

1 More recently, a five-level based scale has been adopted.

2 See the Derivative Free Library (DFL) at http://www.iasi.cnr.it/~liuzzi/DFL
